# Sex-based differences in the distribution of Aujeszky’s disease-seropositive Japanese wild boar

**DOI:** 10.1186/s40813-023-00323-z

**Published:** 2023-06-13

**Authors:** Emi Yamaguchi, Michihiro Takagi, Makoto Osaki, Yoko Hayama, Takehisa Yamamoto

**Affiliations:** 1 Epidemiology Unit, Division of Transboundary Animal Disease Research, National Institute of Animal Health, National Agriculture and Food Research Organization, Kannondai 3-1-5, Tsukuba, 305-0856 Ibaraki Japan; 2 Biologicals Production Office, Department of Animal Disease Control and Prevention, National Institute of Animal Health, National Agriculture and Food Research Organization, Tsukuba, Japan; 3 Virus Group, Division of Infectious Animal Disease Research, National Institute of Animal Health, National Agriculture and Food Research Organization, Tsukuba, Japan; 4 Strategic Planning Headquarters, National Agriculture and Food Research Organization, Tsukuba, Japan

**Keywords:** Wild boar, Aujeszky’s disease virus, Sex-based difference, Spatial clustering, National serosurvey, K-function

## Abstract

**Background:**

Aujeszky’s disease virus (ADV) primarily infects domestic pigs and wild boars, causing the abortion and death of young piglets due to central nervous system disorders. In Japan, the national eradication program for ADV in domestic pigs has been successful in most prefectures; however, concern has been raised regarding ADV-infected wild boars as a source of transmission to domestic pigs.

**Results:**

We assessed the nationwide seroprevalence of ADV among wild boars (*Sus scrofa*) in Japan. Moreover, we determined the sex-based differences in the spatial clustering of seropositive animals. Serum samples were obtained from a total of 1383 wild boars acquired by hunting in 41 prefectures in three fiscal years (April–March in 2014, 2015, and 2017). Seropositivity tests for ADV using enzyme-linked immunosorbent assay, the latex agglutination and neutralization tests showed 29 boars seropositive for ADV (29/1383, 2.1% [95% confidence interval, CI: 1.4–3.0%]), with 28 of these boars originating from three prefectures in the Kii Peninsula (28/121, 23.1% [95% CI: 16.0–31.7%]). The degree of spatial clustering of these ADV-seropositive adult boars in the Kii Peninsula was evaluated using the K-function and data from sera samples of 46 (14 seropositive) male and 54 (12 seropositive) female boars. The degree of clustering among females was significantly higher in seropositive animals than in tested animals; however, such a difference was not observed for seropositive males.

**Conclusions:**

The spatial dynamics of ADV among adult wild boars may be characterized based on sex, and is likely due to sex-based differences in behavioral patterns including dispersal among wild boars.

## Background

Wild boars (*Sus scrofa*) are widely distributed throughout the world, from Eurasia, where they are native, to North and South America and Oceania, where they are non-indigenous [1–3]. In the past several decades, wild boar populations have increased [4, 5], leading to global concerns about crop predation, ecological damage, and disease transmission by wild boars [6–9]. Because their habitats include public and livestock areas [10, 11], various causative agents for livestock and zoonotic diseases may be transmitted between wild boars and domestic pigs, as well as to humans [9, 12, 13]. In Japan, an estimated 870,000 indigenous wild boars lived in most prefectures in 2020 [14]. These animals pose a risk of disease transmission to pig farms including, diseases caused by the classical swine fever virus [15]. Meanwhile, a total of 9.5 million domestic pigs are raised in about 5,000 pig farms throughout Japan. The pigs are mostly kept indoors as an intensive production system. As the population of Japanese boars expands in Japan [3], the risk of infections from wild boars to domestic pigs may also increase. It is necessary to understand the ecology of disease-causing agents in the wild boar population to enable the establishment of an effective control strategy that prevents disease, promotes public health, and enhances livestock hygiene in pig farms.

Aujeszky’s disease virus (ADV), also known as pseudorabies virus or *Suid alphaherpesvirus* 1, is a member of the subfamily *Alphaherpesvirinae* of the family *Herpesviridae*. It primarily infects *Sus scrofa* including domestic pigs and wild boars, causing significant economic losses to pig farmers due to abortion and death of young piglets caused by central nervous system disorders. Mortality and morbidity rates are higher in young piglets and decrease with increasing age [16]. ADV spread worldwide in the 1970s, affecting countries in North America, Oceania, and Europe including the United States, Canada, New Zealand, Germany, and Denmark, ADV has been mostly eradicated from domestic pigs in these countries [16, 17], although serological and virological surveys have revealed the presence of ADV in wild boar populations [18–20]. As the distribution of wild boars increases, the risk of ADV transmission to domestic pig farms also increases due to possible ADV exposure from infected wild boars [18].

In Japan, the first case of Aujeszky’s disease (AD) was detected in domestic piglets in 1981 [21], and by the end of the decade, thousands of ADV-infected domestic pigs had been found in several prefectures [22]. Starting in 1991, a national ADV eradication program was implemented, which involved vaccination using live-attenuated vaccines for domestic pigs. As of June 2021, only one prefecture, Ibaraki Prefecture, was reported as having ADV-infected domestic pig farms [23]. A study by Mahmoud et al. indicated that ADV-seropositive wild boars were present in three prefectures in the western part of Japan [24]. ADV-infected hunting dogs with a history of biting or eating wild boar have been reported in Miyazaki [25] and Oita Prefectures [26], suggesting that wild boars in these prefectures may also be infected. Therefore, infected wild boars can be a source of ADV to domestic pigs in these areas. Wild boars are indigenous to most prefectures in Japan, and their distribution has recently expanded [3]. Moreover, because ADV is lethal to dogs [16], hunting dogs are at risk of contracting ADV by biting infected wild boar, as previously reported [25, 26]. A better understanding of the factors that affect the distribution of ADV-infected boars will help to evaluate the risk of ADV infection in domestic pig farms and hunting dogs.

Previous studies have revealed that various biological factors (e.g., reproductive behavior, feeding habit, and immunosuppression) that differ according to sex can affect the dynamics of infectious diseases among wild mammals [27–29]. In wild boars, experimental and field evidence shows that venereal contact may be the major transmission route for ADV [18, 30, 31]. Although no study has reported that the mortality and severity of AD differ by sex, the behaviors of male and female wild boars differ greatly, which may cause sex-based differences in the dynamics of ADV in the field. Male boars disperse along great distances as they mature [32, 33], whereas females tend to remain near their birthplace within a maternal group comprising female relatives and their offspring [34]. In addition, males roam actively to search for breeding opportunities during winter, i.e., the breeding season [35], during which they mate with several females [34]. A previous field study showed that ADV seroprevalence in females is higher than in males; and changed with the seasons in males among wild swine, which suggested to be caused due to sex-related differences in behaviors [36].

The aims of the present study were to investigate the seroprevalence of ADV among wild boars in Japan and to determine the sex-based differences in the spatial clustering of seropositive animals. We conducted this research to better understand the mechanism of ADV spread among wild boars in Japan and to provide useful information for the planning of control measures for AD and other contact-transmissible diseases.

## Results

Blood samples were collected from 1383 boars in 41 prefectures, consisting of 699, 312, and 372 boars sampled in 2014, 2015, and 2017, respectively (Table 1). Of the total, 312 (22.6% [95% confidence interval (CI): 20.4–24.9%]) and 48 (3.5% [95% CI: 2.6–4.6%]) boars were tested positive for ADV based on the S enzyme-linked immunosorbent assay (ELISA) and gI ELISA, respectively, with 41 boars (3.0% [95% CI: 2.1–4.0%]) testing positive in both ELISAs. Among the 319 boars that tested positive for either ELISA, 29 (2.1% [95% CI: 1.4–3.0%]) tested positive for both the latex agglutination test (LAT) and the serum neutralization test (NT), thus confirming their ADV seropositivity. All boars positive for both LAT and NT were also positive for both ELISAs. The remaining 291 boars that tested positive for either ELISA test were seronegative for ADV. Therefore, it is possible to misdiagnose ADV due to false positive results from nonspecific reactions.

**Fig. 1:**
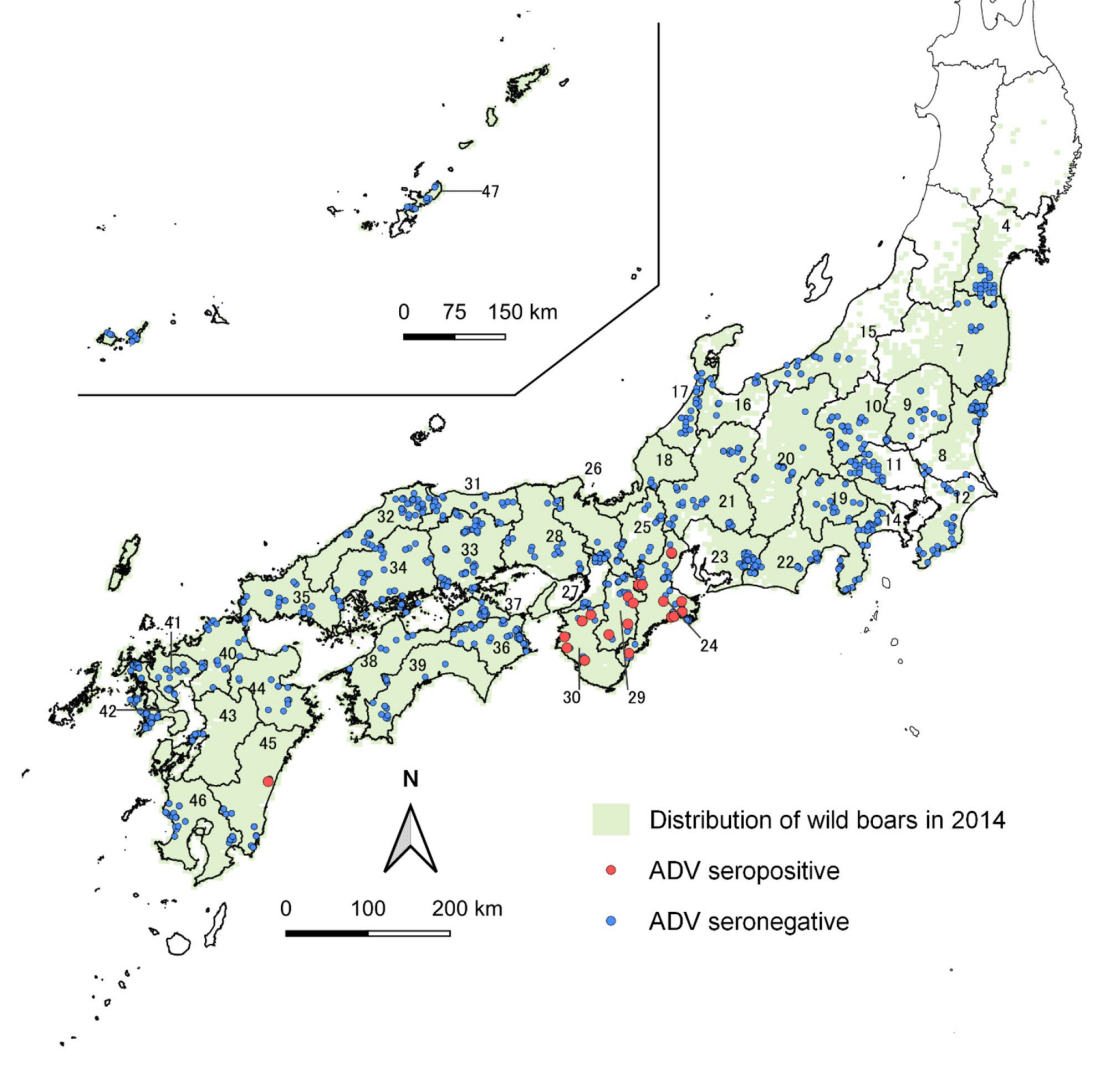
Distribution of Aujeszky’s disease virus (ADV)-seronegative and ADV-seropositive wild boars in Japan in 2014, 2015, and 2017, as determined by a national serosurvey. The numbers on map are Pref IDs in Table 1

**Fig. 2:**
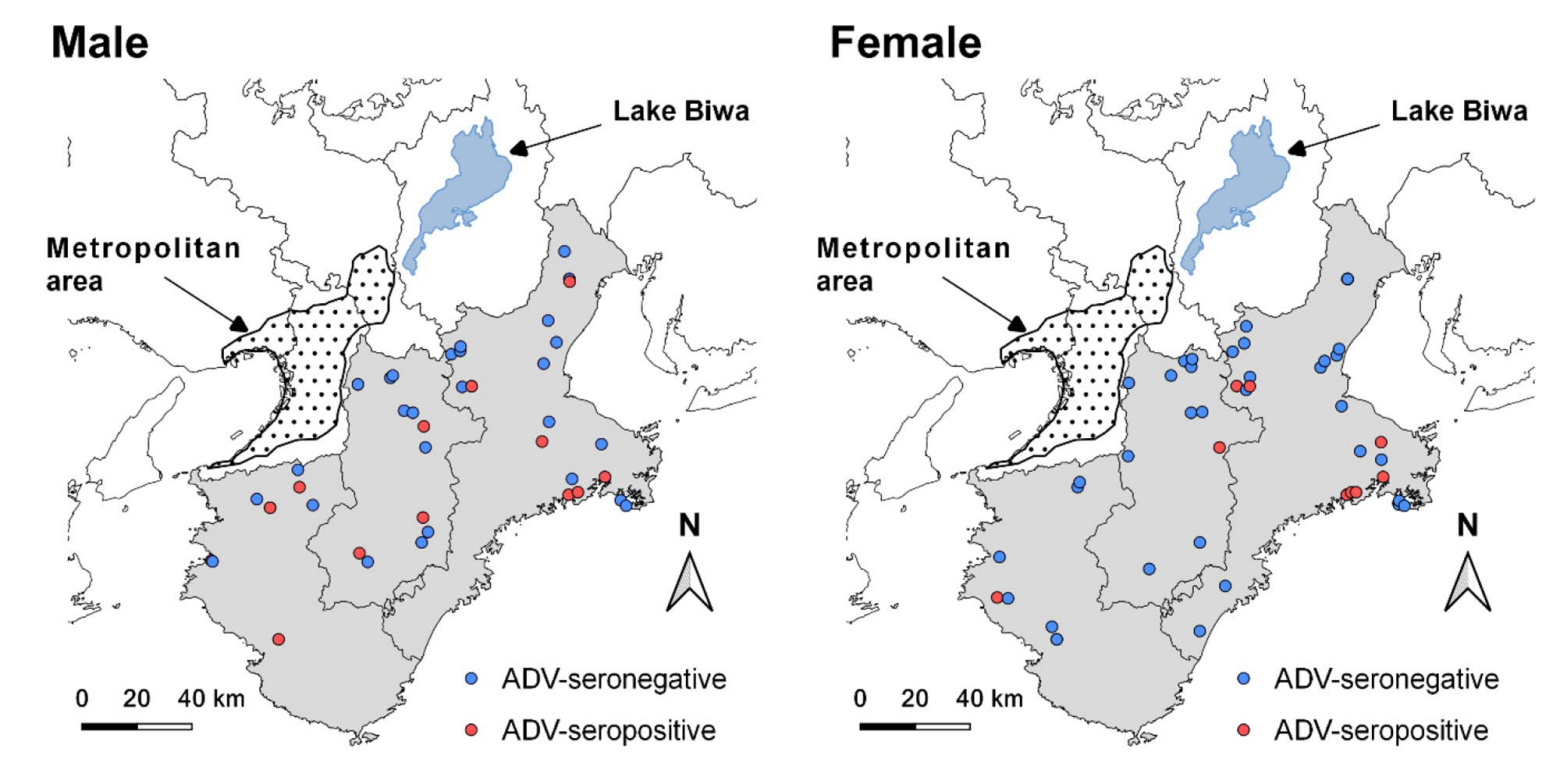
Distribution of male and female adult boars seropositive and seronegative for Aujeszky’s disease virus (ADV) in three prefectures (Mie, Nara, and Wakayama) in the Kii Peninsula (gray area) in 2014 and 2015

Twenty-eight of the 29 seropositive boars were from an area where ADV is endemic and that consists of the neighboring prefectures of Mie, Nara, and Wakayama, all located in the Kii Peninsula, (Table 1; Fig. 1, and Fig. 2). One out of the 29 seropositive boars was from Miyazaki Prefecture. The seroprevalence for ADV among all boars sampled in these prefectures was 23.1% (28/121 [95% CI: 16.0–31.7%]), while among males and females, the seroprevalence rates were 24.6% (15/61 [95% CI: 14.5–37.3%]) and 20.3% (12/59 [95% CI: 11.0–32.8%]), respectively. These seroprevalence rates do not differ significantly (p = 0.73, chi-square test). We were unable to determine the sex of one adult boar. Adult boars had a significantly higher seropositivity than juvenile boars (27/101, 26.7% [95% CI: 18.4–36.5%] vs. 1/20, 5.0% [95% CI: 0.1–24.9%]; p = 0.04, Fisher’s exact test).

**Table 1:** Seroprevalence of Aujeszky’s disease virus among wild boars in 41 prefectures in Japan in 2014, 2015, and 2017. Pref IDs refer to the location of each prefecture in Fig. 1

Pref ID	Prefecture	2014	2015	2017	Total	Prevalence (95% confidence interval)
4	Miyagi	0/0	0/0	0/39	0/39	0% (0–9.03%)
7	Fukushima	0/0	0/0	0/32	0/32	0% (0–10.89%)
8	Ibaraki	0/0	0/0	0/40	0/40	0% (0–8.81%)
9	Tochigi	0/0	0/2	0/29	0/31	0% (0–11.22%)
10	Gunma	0/0	0/45	0/0	0/45	0% (0–7.87%)
11	Saitama	0/0	0/0	0/37	0/37	0% (0–9.49%)
12	Chiba	0/0	0/0	0/34	0/34	0% (0–10.28%)
14	Kanagawa	0/0	0/0	0/36	0/36	0% (0–9.74%)
15	Niigata	0/25	0/0	0/0	0/25	0% (0–13.72%)
16	Toyama	0/17	0/0	0/0	0/17	0% (0–19.51%)
17	Ishikawa	0/0	0/0	0/34	0/34	0% (0–10.28%)
18	Fukui	0/6	0/0	0/0	0/6	0% (0–45.93%)
19	Yamanashi	0/24	0/0	0/0	0/24	0% (0–14.25%)
20	Nagano	0/16	0/0	0/0	0/16	0% (0–20.59%)
21	Gifu	0/38	0/0	0/0	0/38	0% (0–9.25%)
22	Shizuoka	0/24	0/38	0/0	0/62	0% (0–5.78%)
23	Aichi	0/24	0/0	0/0	0/24	0% (0–14.25%)
24	Mie	6/27	11/49	0/0	17/76	22.37% (13.6–33.38%)
25	Shiga	0/20	0/0	0/0	0/20	0% (0–16.84%)
26	Kyoto	0/16	0/0	0/0	0/16	0% (0–20.59%)
27	Osaka	0/0	0/0	0/50	0/50	0% (0–7.11%)
28	Hyogo	0/20	0/0	0/0	0/20	0% (0–16.84%)
29	Nara	4/28	0/0	0/0	4/28	14.29% (4.03–32.67%)
30	Wakayama	7/17	0/0	0/0	7/17	41.18% (18.44–67.08%)
31	Tottori	0/25	0/0	0/0	0/25	0% (0–13.72%)
32	Shimane	0/21	0/43	0/0	0/64	0% (0–5.6%)
33	Okayama	0/19	0/50	0/0	0/69	0% (0–5.21%)
34	Hiroshima	0/33	0/0	0/0	0/33	0% (0–10.58%)
35	Yamaguchi	0/25	0/0	0/0	0/25	0% (0–13.72%)
36	Tokushima	0/26	0/35	0/0	0/61	0% (0–5.87%)
37	Kagawa	0/35	0/0	0/1	0/36	0% (0–9.74%)
38	Ehime	0/15	0/0	0/0	0/15	0% (0–21.8%)
39	Kouchi	0/22	0/0	0/0	0/22	0% (0–15.44%)
40	Fukuoka	0/19	0/0	0/0	0/19	0% (0–17.65%)
41	Saga	0/23	0/0	0/0	0/23	0% (0–14.82%)
42	Nagasaki	0/25	0/50	0/0	0/75	0% (0–4.8%)
43	Kumamoto	0/0	0/0	0/40	0/40	0% (0–8.81%)
44	Oita	0/16	0/0	0/0	0/16	0% (0–20.59%)
45	Miyazaki	1/29	0/0	0/0	1/29	3.45% (0.09–17.76%)
46	Kagoshima	0/36	0/0	0/0	0/36	0% (0–9.74%)
47	Okinawa	0/28	0/0	0/0	0/28	0% (0–12.34%)
	Total	18/699	11/312	0/372	29/1383	2.1% (1.41–3%)

**Fig. 3:**
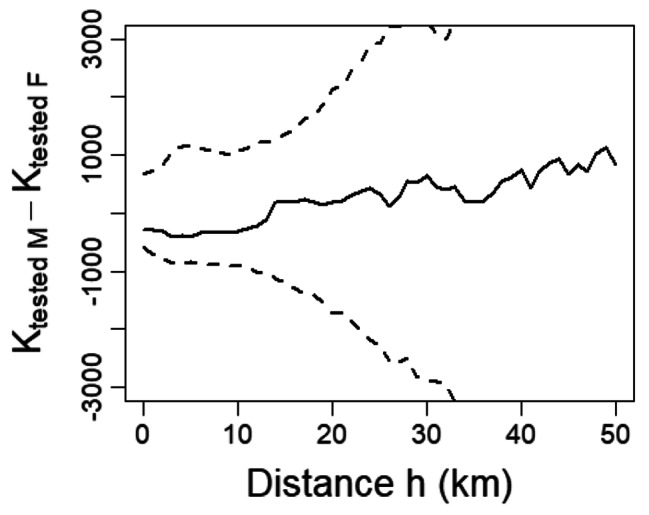
Plot of the empirical K-function K_tested M_ (h) − K_tested F_ (h) (solid line) with the 95% confidence interval (CI) of simulated $\widehat{\text{K}}$_tested M_ (h) − $\widehat{\text{K}}$_tested F_ (h) (dashed line). K_tested M_ (h) and K_tested F_ (h) refer to K-functions of tested male and female boars, respectively. K_tested M_ (h) − K_tested F_ (h) plotted outside of 95% CI of simulated $\widehat{\text{K}}$_tested M_ (h) − $\widehat{\text{K}}$_tested F_ (h) at a scale of distance h indicates that the degree of spatial clustering of tested boars differs significantly based on sex at the km scale

**Fig. 4:**
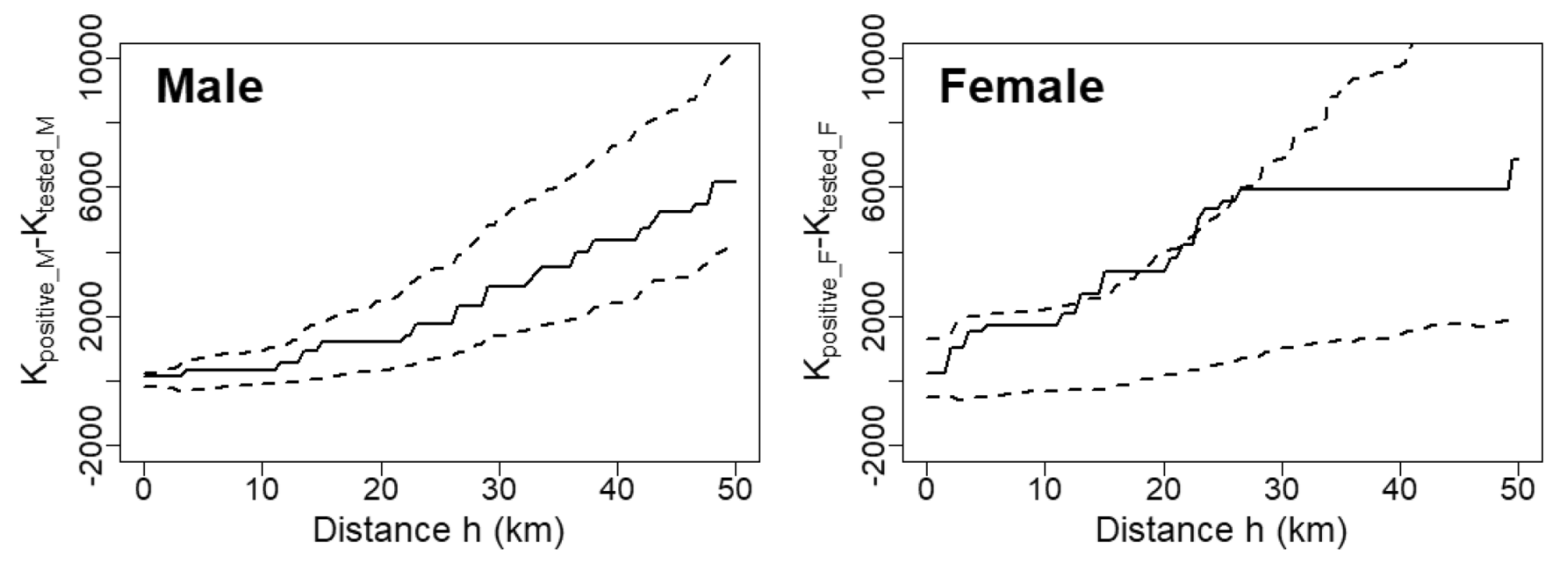
Plot of the empirical K-function (solid line) in adult male (K_positive_M_ (h)) and female (K_posi_F_ (h)) boars with the 95% CI of $\widehat{\text{K}}$_positive M_ (h) (dashed line). Portion of the plot of K_positive M_ (h) that are outside 95% CI of simulated $\widehat{\text{K}}$_positive M_ (h) at a scale of distance h indicates that ADV-seropositive male boars are significantly clustered at the km scale. The significance of spatial clustering in ADV-seropositive boars is identified in the same way

Most of the seropositive boars were from three neighboring prefectures (Mie, Nara, and Wakayama) in the Kii Peninsula; therefore, we regard these prefectures as ADV-endemic areas, and the serum samples of the boars originated from this area were subjected to spatial analysis. Out of 121 tested boars sampled in the endemic area, our spatial analysis considered 100 adult boars, consisted of 46 males (14 seropositive) and 54 females (12 seropositive). Figure 2 shows the spatial distribution of ADV seropositivity among male and female adult boars. Meanwhile, Fig. 3 shows a plot of K _tested M_ (h) − K _tested F_ (h) within the 95% CI of simulated $\widehat{\text{K} }$_tested M_ (h) − $\widehat{\text{K} }$_tested F_ (h), and this plot indicates that the degree of spatial clustering of ADV seropositivity among male and female boars did not differ significantly (Fig. 3). Moreover, in the analyses of the spatial clustering of male seropositive boars, K _positive M_ (h) was plotted within the 95% CI of simulated $\widehat{\text{K} }$_positive M_ (h) (Fig. 4). However, in the analyses of the spatial clustering of female seropositive boars, K _positive F_ (h) was plotted outside the 95% CI of simulated $\widehat{\text{K}}$_positive F_ (h) at distances of approximately 15 and 25 km (Fig. 4).

## Discussion

The results of the current study show the Japan-wide seroprevalence of ADV and sex-based spatial clustering of ADV-seropositive wild boars in 2014, 2015, and 2017. The seroprevalence was 2.1% (29/1383) at the national level, although this figure may be an underestimate because of the low sensitivity of the NT. In addition, because we did not conduct viral detection tests, the detection of ADV viral DNA in seronegative wild boars [20] may also result in underestimation of disease prevalence. Most seropositive boars were localized at the Kii Peninsula (96.6%, 28/29), with 28 out of 121 boars testing seropositive (23.1%), which indicates endemicity of ADV among the boars of the Kii Peninsula.

As shown in Fig. 4, the degree of spatial clustering of ADV-seropositive boars was significantly higher than that of tested boars at distances of approximately 15 and 25 km in females, although such spatial clustering was not detected in males. Meanwhile, a sex-based difference was not observed in the distribution of tested boars. Such sex-based spatial clustering may be attributed to the sex-based differences in behavioral patterns among wild boars. Maternal groups comprising female relatives and their offspring [34] typically have close contacts within the group, which may increase ADV transmission via oral and respiratory routes. As piglets mature sexually, females commonly remain near their birthplace, along with their maternal group, whereas males leave their group and become solitary [34], thus contributing to the spatial clustering of seropositive female boars. In addition, experimental infection studies have shown that ADV is primarily transmitted via venereal contact among cohabiting boars [30]. As male boars roam actively during breeding periods [35] and mate polygamously [34], a single infected male can infect multiple female boars in its roaming area, which will produce a cluster of seropositive females. The distances significant for the spatial clustering in female (i.e., 15 and 25 km) are greater than the radius of the Japanese wild boar home range size, which is 0.9–5.3 km (based on range size of 0.81– 28.5 km^2^) [37, 38], while these distances are similar to the dispersal distance of males, 16.6 km [32]. Accordingly, the dispersal of infected males may contribute to the spatial clustering of seropositive females.

Although more than 20% of wild boars were ADV-seropositive in the Kii Peninsula, we detected no seropositive boar in the prefectures surrounding the peninsula. The Kii Peninsula is surrounded by the metropolitan areas of Osaka and Kyoto, as well as Lake Biwa, the largest lake in Japan (Fig. 2). These geographical barriers may block the translocation of wild boars from the peninsula, thus preventing the spillover of ADV-infected boars to the outside area. Genetic information on the boar populations in and around the Kii Peninsula can help assess the possibility that the localized distribution of ADV-seropositive boars is due to limited boar movement.

In Ibaraki Prefecture, domestic pigs are still infected with ADV; however we detect no wild boar with ADV antibodies in this area. In contrast, ADV has already been eradicated among domestic pigs in the Kii Peninsula [23], where we detected most of the ADV-infected wild boars. Thus, the ADV-endemic areas of domestic pigs and wild boars do not coincide with each other. However, further investigation is required to ensure that domestic pigs remain ADV-free in the Kii peninsula. Venereal contact is the major ADV transmission route among wild boars [18, 30]. Because domestic pigs are mainly raised inside barns in Japan, it is unlikely that wild boars transmit ADV to these animals via direct contact. However, as the wild boar population in this area still presents with ADV, both direct and indirect contact between wild boars and domestic pigs in pig farms should be prevented to maintain the ADV-free status of domestic pigs in the Kii Peninsula.

In the Kii Peninsula and Miyazaki Prefecture, hunters should be conscious about the risk of ADV infecting hunting dogs that come into contact with wild boars. ADV was isolated from a hunting dog that had bitten a wild boar in Miyazaki Prefecture [25]. Moreover, in Oita Prefecture, hunting dogs that ate wild boar died of AD in 2018 [26] (i.e., after the present serosurvey), and we detected no ADV-seropositive boar in this area. The results of this study are derived from surveillance conducted for 1 or 2 years. However, monitoring for ADV infections among wild boars should be continued to identify areas with seropositive wild boars.

A limitation of this study is the non-inclusion of juvenile animals in the spatial analysis, which was done to prevent the influence of maternal antibodies. However, spatial analysis that includes juvenile animals can be helpful to evaluate the possibility of ADV transmission within the maternal group. Evaluating the genetic relationships among tested animals can also help in the assessment of the mechanisms of ADV transmission within and between maternal groups.

## Conclusion

The results of our study show a sex-based difference in the spatial clustering of AD-seropositive adult wild boars in Japan. This difference may be due to the sedentary lifestyle of female boars, combined with and the polygynous mating system of wild boars. Therefore, it is possible that the distribution of other diseases among wild boars can be influenced by sex. Our results will contribute to efforts to understand how disease causing agents such as classical swine fever (CSF) virus and porcine reproductive and respiratory syndrome virus, which are transmitted between individuals, spread within and between groups of wild boars. Wild boars have recently been important carrier of disease-causing agents in humans and domestic animals [9, 12, 13]. Previous studies suggest that wild boars play essential roles in expanding the areas of animals infected with CSF and African swine fever (ASF) viruses, resulting in serious economic losses to pig farmers [39, 40]. Moreover, CSF and ASF infections also caused severe damage to the population and health of wild boars [41, 42].

To establish an effective control strategy against diseases transmitted by wild boars, we need to understand the characteristics of infected wild boars and investigate how certain the biological factors of host animals, such as sex, contribute to infectious disease dynamics among wild boars.

## Methods

### Sample collection

We obtained blood samples from wild boars that were either hunted as game or culled as pests in Japan. Hunting methods included shooting and trapping. The target area for sample collection included prefectures known to be inhabited by wild boars in 2014 [43] (Fig. 1). Samples were collected during three fiscal years (April–March in 2014, 2015, and 2017). Hunting and blood sampling were conducted by hunters who were under a contract between NIAH and the hunter’s association, and who were provided with the necessary sampling tools. Blood was collected in a sterile plastic tube from the carotid artery of boars that had been hunted and killed humanly with gun or electricity. Blood samples were placed on ice packs to maintain their temperature of 4 °C, and the samples were sent to our laboratory within several days after collection. The samples were centrifuged for serum extraction and the serum samples were stored at − 20 °C before performing diagnostic tests. Data from the sampled animals, including sex and body weight, were estimated visually by the hunters, and the kill date and location, as well as hunting method, were recorded. Ages were estimated from body weights (i.e., juveniles: <30 kg, adults: ≥30 kg) [44].

### ADV antibody detection

The serum samples were initially screened using two commercial ELISA kits (the ADV(S) ELISA kit and ADV (gI) ELISA kit [IDEXX Laboratories, Inc., Westbrook, ME]), according to the manufacturer’s instructions. The ADV(S) ELISA kit is based on the Shope strain, and it can detect antibodies to the field and vaccine strains of ADV. Meanwhile, the ADV (gI) ELISA kit can identify the antibodies to the gE antigen of field strains of ADV, excluding the gE-negative vaccine strains, one of which comprises the strain administered in Japan. Samples that tested positive in either of the two ELISA procedures were further analyzed with the LAT (Scientific Feed Laboratory Co., LTD., Japan), using serial dilutions. Samples that agglutinated at more than 40 times dilution were further analyzed using the NT to determine antibody titers, as described in a previous study with some modifications [45]. Briefly, sera were inactivated at 56 °C for 30 min. Each serum sample was diluted twofold by serum-free minimum essential medium (MEM) (Life Technologies, Grand Island, NY) in 96-well U-bottomed tissue culture plates and mixed with an ADV suspension, namely, the Yamagata S81 strain isolated in Japan, containing 4 × 10^3^ TCID_50_/mL. After incubation at 37 °C for 1 h, the diluted serum–virus mixture was added in duplicates to the confluent monolayers of cloned porcine kidney (CPK) cells [46] grown in 96-well flat tissue culture plates. The mixture was allowed to absorb at 37 °C for 1 h. After viral absorption, the cells were washed with phosphate-buffered saline (PBS) and incubated with serum-free MEM for 3–5 days. The plates were monitored for cytopathic effect (CPE) via microscopy. In the present study, several days elapsed prior to serum extraction, which caused hemolysis in some samples; and ELISAs performed on hemolyzed sera may result in false positive due to nonspecific reactions [47]. Therefore, LAT and NT were also conducted, and only samples that were positive in both tests were considered ADV-seropositive. Seroprevalence was summarized according to prefectures and plotted on a map at an individual level. Because ADV is latent in the trigeminal ganglia of infected hosts, and these animals have life-long antibody reactions [16], seropositive boars were considered infected with ADV.

*Detection of sex-based differences in the spatial clustering of ADV-seropositive boars*.

The association between sex and age to seropositivity for ADV among boars was evaluated using the chi-square test or the Fisher’s exact test. Sex-based differences in the distribution of ADV-seropositive wild boars were then assessed based on the degree of spatial clustering of male and female seropositive animals. Spatial clustering analysis considered only adult boars to prevent the effects of maternal antibodies among juveniles [48]. We first examined for sex-based differences in spatial clustering in tested boar distribution, because a sex-based difference in the clustering of seropositive individuals may be biased if the clustering of tested boars differed according to sex.

Ripley’s K-function analysis evaluates the degree of spatial clustering in a point pattern by counting the average number of neighbors of each point within a certain distance h [49]. The K-function was estimated as

1 $$\widehat{K}\left(\text{h}\right)=\frac{{\widehat{\lambda }}^{-1}}{n}\sum _{i=1}^{n}\sum _{{j=1}_{i\ne j}}^{n}\frac{{I}_{h}\left({d}_{ij}\right)}{{w}_{ij}}, h>0$$,

where h is the given distance, n is the number of tested or seropositive boars, d_ij_ is the Euclidian distance between animals i and j, and λ is the intensity of events (i.e., the average number of tested or positive boars per unit area). I_h_ (d_ij_) is a function that is equal to 1 if d_ij_ < h; otherwise, it is equal to 0. w_ij_ is the proportion of the study area within the circle of radius d_ij_ centered at point i. In this study, distance h ranged from 0.5 to 50 km by 0.5 km. By comparing K-functions between empirical and simulated events, this analysis generates results that can indicate whether the empirical events are spatially clustered at a certain distance.

Sex-based difference in the spatial clustering of the tested samples was assessed by evaluating the difference between the K-function of male (K_tested M_ (h)) and female (K_tested F_ (h)) animals. The significance of the difference in spatial clustering between sexes was evaluated by comparing empirical K _tested M_ (h) − K_tested F_ (h) and simulated $\widehat{\text{K}}$_tested M_ (h) – $\widehat{\text{K}}$_tested F_ (h) at each distance h [50, 51]. Simulated $\widehat{\text{K} }$_tested M/F_ (h) was generated by taking 10,000 sample sets of randomly selecting male and female samples with the same number of empirical data in each sex.

The degree of spatial clustering of ADV-seropositive boars was evaluated. Since the hunting locations of ADV-seropositive boars are dependent on the locations of tested boars, the degree of spatial clustering of ADV-seropositive boars was assessed by comparing the K-function of ADV-seropositive boars (K_positive_ (h)) of each sex to that of tested boars (K_tested_ (h)). The significance of spatial clustering was evaluated by comparing empirical K_positive_ (h) and simulated $\widehat{\text{K}}$_positive_ (h) values for each distance h for each sex. The simulated $\widehat{\text{K}}$_positive_ (h) was generated by using 10,000 sample sets of randomly selected ADV-seropositive samples with the same number of empirical data for each sex. The differences between the empirical and simulated values of each function with 95% CI were plotted. All empirical values that were lower or higher than the 95% CI were considered significantly different in the test of the degree of spatial clustering. Geographical and statistical analyses were performed using QGIS version 3.10.1 and R version 4.0.2 [52], respectively. The K-function was calculated using the splancs R-package [53].

## Data Availability

The datasets used during the current study are the corresponding author on reasonable request.

## Abbreviations

ADV: Aujeszky’s disease virus
AD: Aujeszky’s disease
ASF: African swine fever
CI: Confidence interval
CPE: Cytopathic effect
CPK: Cloned porcine kidney
CSF: Classical swine fever
ELISA: Enzyme-linked immunosorbent assay
LAT: Latex agglutination test
NT: Neutralization test
MEM: Minimum essential medium
PBS: Phosphate-buffered saline
